# Myeloid-Derived Suppressor Cells as a Therapeutic Target for Cancer

**DOI:** 10.3390/cells9030561

**Published:** 2020-02-27

**Authors:** Andrew M. K. Law, Fatima Valdes-Mora, David Gallego-Ortega

**Affiliations:** 1 Tumour Development Group, The Kinghorn Cancer Centre, Garvan Institute of Medical Research, Sydney, NSW 2010, Australia; 2 Histone Variants Group, Garvan Institute of Medical Research, Sydney, NSW 2010, Australia; 3 St. Vincent’s Clinical School, Faculty of Medicine, University of New South Wales Sydney, Sydney, NSW 2052, Australia

**Keywords:** Myeloid derived suppressor cells, tumour microenvironment, immunotherapy, immune system, immune checkpoint inhibitors

## Abstract

The emergence of immunotherapy has been an astounding breakthrough in cancer treatments. In particular, immune checkpoint inhibitors, targeting PD-1 and CTLA-4, have shown remarkable therapeutic outcomes. However, response rates from immunotherapy have been reported to be varied, with some having pronounced success and others with minimal to no clinical benefit. An important aspect associated with this discrepancy in patient response is the immune-suppressive effects elicited by the tumour microenvironment (TME). Immune suppression plays a pivotal role in regulating cancer progression, metastasis, and reducing immunotherapy success. Most notably, myeloid-derived suppressor cells (MDSC), a heterogeneous population of immature myeloid cells, have potent mechanisms to inhibit T-cell and NK-cell activity to promote tumour growth, development of the pre-metastatic niche, and contribute to resistance to immunotherapy. Accumulating research indicates that MDSC can be a therapeutic target to alleviate their pro-tumourigenic functions and immunosuppressive activities to bolster the efficacy of checkpoint inhibitors. In this review, we provide an overview of the general immunotherapeutic approaches and discuss the characterisation, expansion, and activities of MDSCs with the current treatments used to target them either as a single therapeutic target or synergistically in combination with immunotherapy.

## 1. Introduction

The immune system is a complex and dynamic system that operates through an intricate network of cellular interactions and transient functional states. It is involved in various biological activities and is the sine qua non for natural defense of the human body against pathological processes. In cancer progression, the immune system plays a pivotal role where immune cells infiltrate tumours, co-evolving and cooperating with cancer cells to create an inflammatory and immunosuppressive microenvironment to facilitate tumour growth and dissemination. 

In the early stages of carcinogenesis, immunologically vulnerable neoplasms are contained and abrogated by immune cells upon detection by immunosurveillance, a process where the immune system inhibits aberrant cell growth. The elimination of immunogenic neoplasms creates a selective pressure that drives the propagation of non-immunogenic clones with adapted mechanisms for immune evasion and survival. Paradoxically the immunosurveillance process against tumour cells promotes the immunoselection of poorly immunogenic variants. The continuous cycle of immune selection for resistant cancer variants leads to tumour escape through multiple mechanisms, including reduced expression of tumour-associated antigens and co-stimulatory molecules, including major histocompatibility complex (MHC) [1]. Tumour cells can also hijack mechanisms that confer survival advantages by increasing proliferation and/or reducing apoptosis [2]. This paradigm of cancer “immunoediting” describes the evolution and selection of cancer cells to develop clinically relevant tumours.

The development, survival, and spread of cancer cells involve a myriad of complex interactions between cancer and immune cells; in which immune cells are involved in both pro-tumourigenic and anti-tumourigenic roles [3,4,5,6]. The diverse immune milieu that exists within the tumour microenvironment (TME) secretes various signals that orchestrate the development and progression of cancer through the selection of pro-tumourigenic characteristics such as bypassing apoptotic pathways, immunoevasion, and maintaining inflammation and angiogenesis [3]. As the TME develops and evolves, immunosuppressive cells such as T-regulatory cells (Treg) and myeloid derived suppressor cells (MDSCs) are co-opted to inhibit the proliferation and activity of killer T cells; thereby promoting tumour progression and metastasis. On the other hand, the immune system can be stimulated to elicit an immune response that targets the tumour for eradication. Thus, the main theory of immunotherapy resides on the plasticity of the immune system and its capacity to be re-educated into restoring a potent anti-tumourigenic response. Thus, immunosuppressive cells within the TME have become a major target for improving the efficacy of immunotherapy, and multiple therapeutic strategies have been developed in the last few years.

In this review, we examine the phenotypic characteristics of MDSCs, their immunosuppressive functions and the mechanisms they employ to suppress anti-tumour response, how a pro-inflammatory TME drives MDSC expansion, and current treatments that are used to target MDSCs. Finally we discuss the synergistic treatments of combining immune checkpoint inhibitors with MDSC targeting.

## 2. Immunotherapy Against Cancer

Over the last few years, there has been increasing interest in developing cancer therapeutics that target different aspects of the immune system. With the common aim of re-educating or re-activating the immune milieu to produce a potent immune rejection of cancer cells, different strategies have been established or are under intense development.

### 2.1. Cancer Vaccines

Cancer vaccines are generated using different approaches. They are accomplished by exposure to cancer cells, cell lysates, RNA/DNA, or engineered-viral tumour antigens, but most commonly developed through tumour-associated antigens (TAA) [7,8]. These vaccines can be used for prevention—such as the FDA-approved hepatitis B virus (HBV) vaccine for liver cancer and human papillomavirus (HPV) vaccine for cervical cancer [9,10]—or therapeutically to regulate the progression of existing tumours. TAA are often used in cancer vaccine development and can be primarily characterised as either (i) differentiation antigens: tissue-specific proteins that are expressed in the tumour and the analogous normal tissue, but are aberrantly expressed in the tumour due to hyperproliferation of particular cell type (e.g. PAP); or (ii) overexpressed antigens: highly expressed proteins in tumours due to malignancy, but have a baseline expression in healthy tissue (e.g. Her2/neu) [11,12]. As such, the TAA selected for the vaccine is critical to ensure appropriate specificity and induction of T-cell activity against the antigen in tumours [13]. Unfortunately therapeutic vaccines have not demonstrated as successful clinical efficacy as other immunotherapy efforts such as CAR T-cell therapy or checkpoint inhibitors [14]. This poor outcome can be attributed to diverse antigen expressions due to tumour heterogeneity, low levels of tumour-infiltrated lymphocytes (TILs), and the evolution of different immunosuppressive mechanisms that have developed over the course of the cancer progression. Dampening the immune response through expansion of immunosuppressive cells, expression of inhibitory cytokines and proteins, and angiogenesis have all contributed to the disappointing clinical result [14,15]. 

### 2.2. Monoclonal Antibodies

Similar to cancer vaccines, monoclonal antibodies target specific antigens dysregulated in cancer cells to either elicit an immune response against the tumour or for direct drug delivery [16]. The monoclonal antibodies can be unconjugated, conjugated with chemotherapy or radiolabelled, or bispecific [8,17]. Unconjugated antibodies mark cancer cells for immune destruction and can also be used to inhibit antigen functions; for example, targeting HER2 overexpression by Trastuzumab in the treatment of breast cancer can reduce cancer cell proliferation via HER2 degradation and induce antibody-dependent cellular cytotoxicity [18,19]. 

### 2.3. Adoptive T cell Therapy

**Figure 1:**
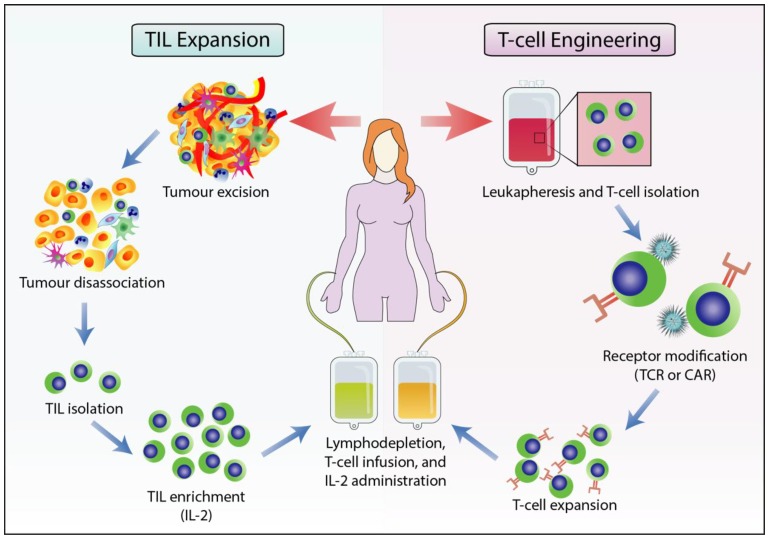
Workflow of adoptive T-cell therapy using TILs or receptor modified T-cells. Adoptive T-cell therapy can improve anti-tumour response by expanding TIL populations extracted from patient tumour (left), or genetically modifying the TCR or generating a chimeric antigen receptor (CAR) (right). With TIL expansion, the patient tumour is surgically resected and the TILs are isolated and expanded ex vivo. The TIL populations are then further increased through a Rapid Expansion Protocol before they are intravenously infused back into the lymphodepleted patient. For the genetic modification of T-cell, the TCR and CAR-T therapy extracts T-cells from the peripheral blood via leukapheresis and are transduced with viral vectors to express a modified TCR or CAR. In both approaches, the patient is lymphodepleted with cyclophosphamide before T-cell infusion and is administered with IL-2 to improve treatment efficacy and longevity.

Adoptive cell therapy (ACT) enhances anti-tumour rejection by infusing patients with enriched and modified T cells via two primary methods: i) expanding isolated TILs or ii) genetic modification of peripheral blood T cells to enhance tumour cell recognition (Figure 1) [20]. 

In the first method, TILs are cultured from a resected tumour surgically extracted from the patient. TIL populations are rapidly expanded ex vivo with high levels of interleukin-2 (IL-2). The patient is preconditioned through a nonmyeloablative lymphodepletion using cyclophosphamide before the TIL cultures are intravenously infused back into the patient. This preconditioning regimen and the administration of subsequent IL-2 has been shown to increase the duration and clinical response of TIL therapy [21]. This form of ACT has resulted in an objective response rate of up to 50% in patients with metastatic melanoma [22,23], and has extended its application to other forms of solid tumour cancer, including breast cancer [24].

The second approach of ACT uses T-cells extracted from the peripheral blood via leukapheresis, which are then genetically modified to improve tumour cell recognition. This is done by transducing T-cells with retroviral or lentiviral vectors to highly express novel TCRs that target specific TAAs [25,26]. To circumvent immune evasion in cancer cells by MHC aberrant expression or reduction, T-cells can be alternatively modified to express chimeric antigen receptors (CAR) [27]. CAR T-cells function similarly to TCR-modified T-cells but can recognise TAA in an MHC-independent manner [26]. CAR T-cell therapy has reported significant clinical response, with up to 90% complete remission rates in acute lymphoblastic leukemia targeting the B-cell antigen CD19 [28]; it has also shown high efficacy in the treatment of leukemia using CD22-directed CAR T-cells [29]. Despite successful clinical trials, a major challenge in adoptive immunotherapy remains in targeting tumour-specific antigens as most antigens expressed on tumours are also present on normal tissue [30]. This incites on-target toxicity through T-cell targeting of shared antigens on both tumours and healthy tissue. As such, other T-cell modification strategies that employ bispecific antigen detection systems or T-cell redirection are currently under study. For example, inhibitory chimeric antigen receptor (iCAR) or tandem chimeric antigen receptors (TanCAR) are receptors that can be engineered onto T-cells to enhance their cytotoxicity and specificity to tumour antigens (reviewed in [31,32]).

### 2.4. Immune Checkpoint Inhibitors

**Figure 2:**
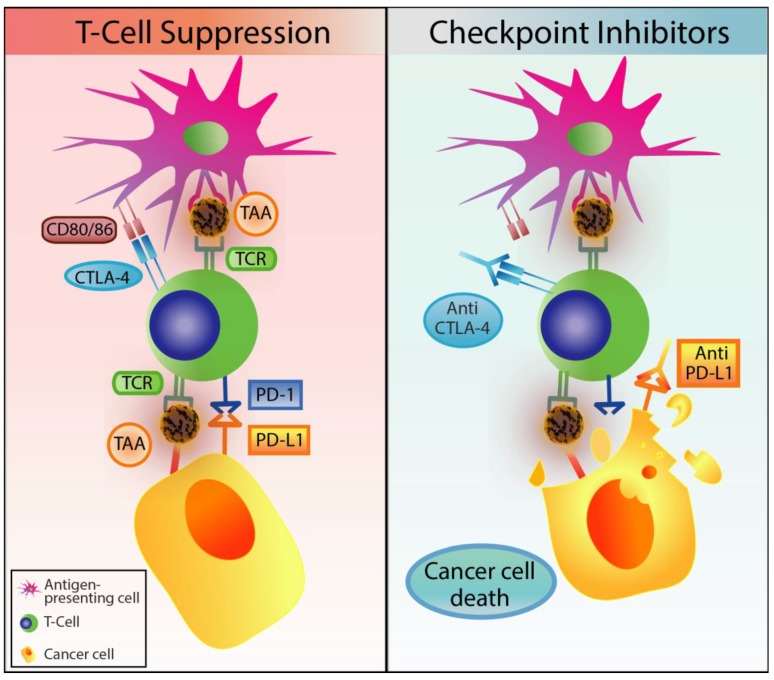
Immune checkpoint blockade of T-cell activity and mechanism of action of checkpoint inhibitors. The immune checkpoints regulate T-cell activity and are crucial for maintaining self-tolerance. However, in cancer, the endogenous T-cell immune checkpoints, CTLA-4 and PD-1, inhibit T-cell activity when bound to their ligands, CD80/86 (antigen-presenting cells) and PD-L1 (cancer cells), respectively. Treatments with checkpoint inhibitors can disrupt this regulatory interaction allowing T-cell cytotoxic activity against cancer cells.

Checkpoint inhibitors as immunotherapy had elicited an impressive response in the treatment of melanoma and lung cancer; with so much potential, this type of immunotherapy was considered as Breakthrough of the Year in 2013 by the journal *Science* [33] and awarded the Nobel Prize in Medicine 2018 [34]. Immune checkpoint pathways are co-inhibitory signals that are manipulated during cancer to downregulate the immune response. Immune checkpoint inhibitors, such as Ipilimumab and Nivolumab, target the checkpoint pathway of cytotoxic T cells (CTL) though cytotoxic T-lymphocyte-associated protein 4 (CTLA-4) and programmed death 1 (PD-1), respectively. CLTA-4 is a receptor that is expressed on the surface of T cells and inactivates T cell activity by competing against CD28 to bind to the two T cell activation antigens CD80 and CD86, found on the surface of antigen-presenting cells (APC). In addition, the PD-1 receptor is also found on T cells, where, upon binding to the ligand PD-L1, induces a conformational change to an inactive and dysfunctional state [35]. As such, by targeting these two checkpoint pathways, the baseline of T cell activity can be restored to reactivate tumour immunosurveillance (Figure 2). 

Despite the therapeutic success of checkpoint inhibitors for some cancer types, a primary challenge of this strategy for widespread anti-cancer application remains the low TILs presented by patients of many cancer types. Since checkpoint inhibitors rely primarily on pre-existing TILs, patients with low immunogenic tumours will likely be non-responsive to checkpoint inhibitor therapy [36]. A clear example is breast cancer, where only the genomically unstable Triple Negative Breast Cancer (TNBC) has shown limited responses to checkpoint inhibitors [37,38]. As such, the success rates of immunotherapy are often unpredictable, having significantly variations with different cancer types and even within cohorts consisting of the same malignancy, for example in advanced ER+ breast cancer [39,40]. However since checkpoint inhibitors interfere with natural T-cell regulatory mechanisms, they can also lead to activation of autoreactive T-cells, resulting in autoimmune or autoinflammatory side-effects termed “immune-related adverse events” (irAEs) [41].

The discrepancy in patient response demonstrates critical limitations in our knowledge of immunotherapy: (1) why immunotherapy works for some patients and not others; (2) why the frequency and severity of irAEs varies in patients, though different dosing regimens and strategies of immunotherapy combination are currently being investigated to reduce toxicity [42]; and (3) how the immunosuppressive TME plays an extensive role in the efficacy of these types of immunotherapy. These limitations have driven more research on the interplay of the immune system during the carcinogenic process. In this regard, new strategies to overcome the immunosuppressive TME have been a major focus. These strategies include: (1) increasing TIL levels by abolishing the endothelial barrier, which prevents T-cell infiltration; forcing T-cell accumulation at the adjacent stroma and reducing their traffic into the tumour [43]; and (2) by eliminating the immunosuppressive TME to stimulate anti-tumour immunity [44]. Immune cells such as tumour-associated macrophages (TAM), MDSC, and Tregs can function to stimulate angiogenesis through secretion of VEGFA and PGE2, thus creating an endothelial barrier [45,46]; and promote immunetolerance via CTL and NK cell suppression [47,48,49,50]. As such, targeting these pro-tumourigenic immune cells to alleviate the immunosuppressive microenvironment may be key to improving the efficacy of the aforementioned treatment strategies. An immunosuppressive target that has gained increasing attention in the last few years is the MDSC. The accumulation of these myeloid progenitors in patients has been attributed to resistance against checkpoint inhibitors and may potentially be used as a predictive marker for treatment success [51].

## 3. Classification and Function of Myeloid-Derived Suppressor Cells

**Figure 3:**
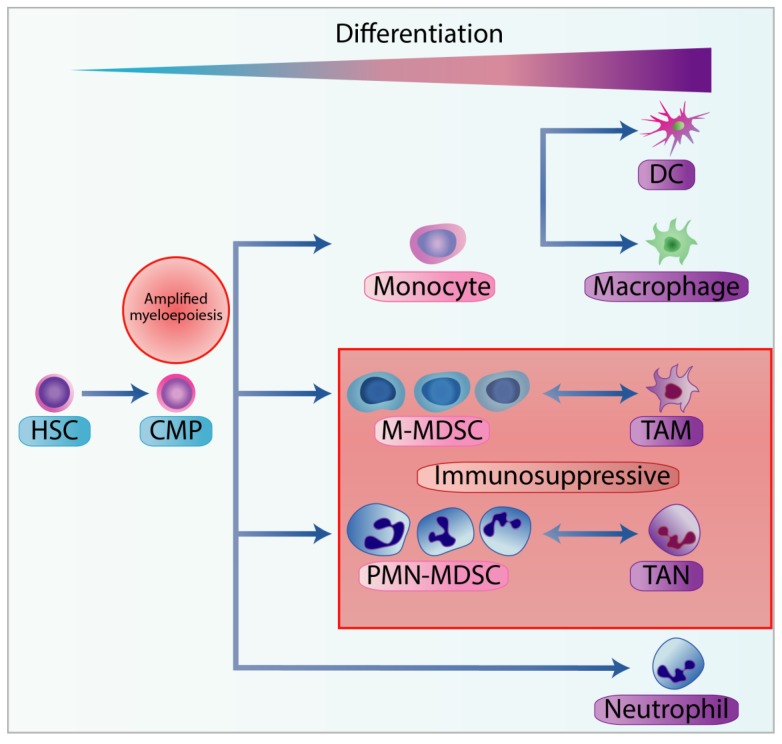
Stages of myelopoiesis differentiation in cancer. Myelopoiesis is amplified during chronic inflammation to assist tumour progression and dissemination. The hematopoietic stem cells (HSC) differentiate into the common myeloid progenitor (CMP), which can further differentiate through the hematopoietic system. In physiological conditions, CMP can differentiate into neutrophils or into monocytes, and subsequently into dendritic cells (DC) or macrophages. However, with chronic inflammation, pro-inflammatory cytokines can skew the monocytopoiesis of CMP into monocytic-myeloid-derived suppressor cells (M-MDSC) and tumour-associated macrophages (TAM), and granulopoiesis into polymorphonuclear myeloid-derived suppressor cells (PMN-MDSC) and tumour-associated neutrophils (TAN).

MDSCs are comprised of a heterogenous immature myeloid cell (IMC) population in various states of transcriptional activity and differentiation [52]. The myeloid lineage is expanded during pathological conditions, where emergency myelopoiesis increases the production of myeloid leukocytes in the bone marrow to eradicate potential threats such as pathogens, tissue damage, chronic inflammation, and cancer [53]. Chronic inflammation in cancer is induced in the TME through the expression of pro-inflammatory cytokines, such as PGE2, GM-CSF, G-CSF, M-CSF, SCF, S100 proteins, VEGF, TGFβ, and TNFα. The combinatorial effects of these cytokines can skew the differentiation in favour of MDSCs and perturb the maturation of myeloid cells; this can create a spectrum of IMC that is morphologically analogous to granulocytes and monocytes but can vary in the presence of particular cell surface markers (Figure 3) [54,55].

In addition to chronic inflammation, the amplified state of myelopoiesis is also manipulated in cancer to promote tumour progression and dissemination [56,57,58,59]. MDSCs within the bone marrow are recruited to the peripheral lymphoid organs and the tumour site by growth factors secreted by cancer cells; this, in turn, promotes tumourigenesis via different mechanisms by: permitting immunoevasion by inducing NK cell and T-cell anergy; remodelling the TME to promote tumour growth; creating and establishing a metastatic niche for cancer dissemination; inducing epithelial-mesenchymal transition (EMT) and mesenchymal-epithelial transition (MET) transition to facilitate tumour progression and metastasis; promoting angiogenesis; and improving tumour cell survival through their immunosuppressive activities [60,61,62,63,64,65]. As such, MDSCs actively contribute to an immune-tolerant TME and impede the efficacy of cancer immunotherapies. In a meta-analysis study conducted by Zhang and colleagues, abundance of MDSC in patients with solid tumours has been correlated with poorer prognosis and overall survival [66].

### 3.1. Classification of MDSC

MDSCs can be broadly categorised into two groups: polymorphonuclear (PMN-MDSC) and monocytic (M-MDSC). In mice, M-MDSCs are mononuclear and express high Ly6C and low or absent Ly6G (CD11b+Ly6Glow/−Ly6C+), whereas PMN-MDSC consist of multilobed nuclei that are neutrophil-like and express Ly6G and low Ly6C (CD11b+Ly6G+Ly6Clow) [59]. PMN-MDSCs and M-MDSCs are phenotypically and morphologically analogous to neutrophils and monocytes, respectively, and thus using only phenotypic criteria to identify MDSCs is insufficient. The Ly6G and Ly6C markers are murine-specific and no orthologues exist in humans. In contrast, human MDSCs are identified based on myeloid cell markers CD11b+, CD33+, HLA-DRlow/− and negative for lineage-specific antigen (Lin−) and the same two MDSC subsets can be characterised by CD11b+CD33+HLA-DR−/CD14+CD15− for M-MDSC and CD11b+CD33+HLA-DR−/CD14-CD15+ for PMN-MDSC [56,67]. However, due to the heterogenous nature of the MDSC populations both biochemically and functionally, distinct subtypes of MDSC have been isolated from different types of cancer, and combinations of molecular markers used to identify MDSC populations can vary based on disease context (Table 1 and Table 2) [68,69,70]. MDSCs have been also identified by using different sets of markers such as CD11b+CD33+CD34+ [71], Lin−/Low HLA-DR−, CD33+CD11b+ [72], and CD14+HLA-DR-/Low [73]. Thus, there is still no established consensus on the combination of markers that should be used for determining MDSC presence in tumours [74]. The proportion of infiltrated M-MDSC and PMN-MDSC varies with tumour type and progression of the disease. For example, in breast cancer, PMN-MDSC is the predominantly expanded population compared to M-MDSC [52]. Clinically, MDSC sub-classification is essential, as these subsets are functionally different, presenting different mechanisms of activation and immunosuppression.

### 3.2. MDSC Recruitment and Pro-Tumorigenic Activation

**Figure 4:**
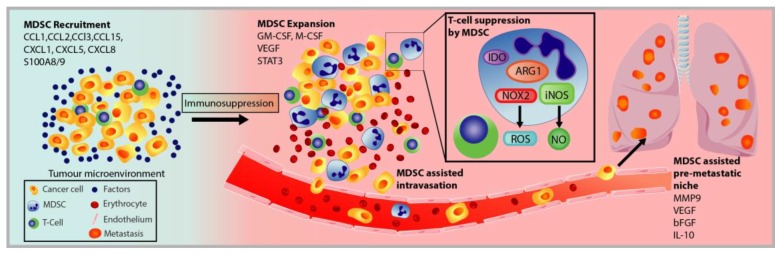
Schematic of MDSC recruitment and role in cancer progression and metastatic spread. MDSC are recruited to the tumour site by the same factors that mobilise neutrophils and monocytes. Within the tumour microenvironment, the MDSC population expands and exerts their immunosuppressive functions to induce T-cell and NK cell anergy through different mechanisms, such as through the enzymes IDO, ARG1, iNOS, and NOX2. MDSC can also assist in cancer cell dissemination through the promotion of angiogenesis, EMT and MET transition, and secretion of tumourigenic factors.

The recruitment and expansion of MDSCs to the primary and metastatic tumour sites are regulated by a combination of tumour-derived factors secreted by the TME and cancer cells, and it continuously evolves and develops (Figure 4). These factors can be categorised as (1) trafficking signals used by cancer cells to induce MDSC expansion and recruitment into the tumours, and (2) activation signals of MDSCs secreted by tumour stroma and T-cells [61].

PMN-MDSC and M-MDSC recruitment to tumours is essentially governed by the same factors that regulate the migration of neutrophils and monocytes. M-MDSC and inflammatory monocytes are recruited to tumours through a CCL1, CCL2, and CCL5-induced chemokine cascade that is propagated by cancer cells and has been found to be retained within primary tumours by CCL3 produced via CCR-2 activated mechanism in metastasis-associated macrophages [152,153,154]. Similarly, PMN-MDSC and neutrophils are also recruited to tumours by CCL2 and CCL3 [155,156]. Hypoxia at the primary tumour site has also been linked to the recruitment and activation of MDSCs to promote an immunosuppressive environment and the establishment of a pre-metastatic niche in secondary organs [65].

During metastasis, MDSC populations are recruited in the pre-metastatic lungs in mice with mammary carcinoma through the chemoattractants CXCL1, CXCL2, CXCL5, and S100A8/9 [157]. It is believed that these MDSCs arrive initially to create a pre-metastatic niche to condition distal organs for tumour dissemination [157]. MDSCs promote cancer cell invasion by establishing an immune-tolerant and inflammatory environment through the downregulation of IFNγ, overexpression of inflammatory cytokines, and inducing leaky vasculature by expressing matrix metalloproteinase 9 (MMP9) and other remodelling factors to diminish the integrity of the extracellular matrix (ECM) and the basal membrane [62]. Cancer cells are then recruited to the metastatic site via TNFα, CXCL2, TGFβ, and S100A8/9 [158]. MDSCs also express factors, such as TGFβ, HGF, and IL-6, to facilitate EMT-MET transition in cancer cells [157]. 

The chemoattractants expressed by cancer cells to recruit MDSC are ubiquitous in different types of cancers. As such, therapeutic blockade targeting chemokine receptors will be a more effective target than targeting the ligands themselves as a single receptor may interact with multiple chemokines [131]. For example, CXCR2 is highly upregulated in tumour recruited neutrophils and MDSCs, and abrogation of CXCR2 signalling significantly improved T-cell infiltration and extended survival in both cancer patients and mice models [118], especially in combination with other immune checkpoint blockades such as PD-1 treatments [150,159].

MDSC activation and survival are regulated by the signal transducer and activator of transcription (STAT) family, such as STAT1, STAT3, STAT6, and NFkB [47]. Cancer cells, tumour-associated stromal cells, and activated T-cells play a role in initiating these signalling pathways involved in MDSC activation via the expression of TLR4, IL-1β, TGFβ, IFNγ and IL-4 [67]. The transcription factor STAT3 has been associated as one of the main drivers of MDSC expansion, and together with other factors such as GM-CSF, M-CSF and VEGF, contribute to the increase of MDSC levels within the tumour [67]. Since the downstream targets of STAT3 are primarily affiliated with genes that regulate proliferation and pro-survival, such as survivin, BCL-XL, and cyclin D1, it is unsurprising that upregulation of STAT3 facilitates MDSC expansion by inhibiting IMC differentiation into mature myeloid cells and increasing proliferation [67,160]. In addition, STAT3 also upregulates the S100A8/9 pro-inflammatory proteins, which drive a feedback loop in the migration and result in accumulation of MDSCs. S100A8/9 are found ubiquitously in most tumours and increased S100A8/9 has also been shown to prevent the differentiation of myeloid progenitor cells and deactivation of T-cell in breast, ovarian, and gastric cancer [161,162,163]. Thus, S100A8/9 has been implicated as playing a vital role in the link between inflammation and immunosuppression [47].

Downregulation of STAT3 has also been previously reported to drive the pathological differentiation of M-MDSCs into M2-like TAMs [164]. Some studies have indicated that MDSC exposure to a hypoxic TME allows CD45 protein tyrosine phosphatases (PTP) or hypoxia-inducible factor (HIF1α) to induce inflammatory monocytes and MDSC differentiation into TAM, which permits their immunosuppressive functions to be exerted [164,165,166]. However, MDSC differentiation still remains unclear and other studies have suggested that M-MDSCs and PMN-MDSCs may have distinct routes of pathological differentiation within the TME.

### 3.3. Immunosuppression of MDSC

Activated MDSCs have an array of mechanisms that are utilised to create an immunosuppressive microenvironment, inducing anergy in NK cells and in CD4+ and CD8+ T-cells, and thus promoting immunetolerance in cancer. These include metabolic-based mechanisms that deplete essential amino acids for T-cell activity and proliferation, and mechanisms based on the secretion of specific factors involved in immunesuppression, such as the expression of PD-L1 by MDSCs [167]; expression of immunosuppressive cytokines such as IL-10 and TGFβ [114,168]; and recruitment of Tregs via expression of CD40 by MDSCs [87]. 

Metabolically, MDSCs can sequester cysteine and compete against T-cell [169]. This amino acid is essential for T-cell activation and proliferation and cannot be synthesised de novo by T-cells; as such, T-cell function is reliant on exogenous supplies of cysteine [92]. The accumulation of MDSCs within the TME consumes and reduces the level of environmental cysteine, resulting in T-cell inhibition via cysteine depletion [169]. In addition, MDSCs can further deplete the TME of essential amino acids by catabolising L-arginine and L-tryptophan. L-arginine is metabolised by the expression of arginase-1 (ARG1) and inducible nitric oxide synthase (iNOS) [170]. The high expression of ARG1 and iNOS in MDSCs depletes L-arginine by catabolising it into L-ornithine and urea (ARG1) or into NO (iNOS) [47,171]. L-arginine starvation and production of NO within the TME is detrimental for T-cell function and proliferation, as it can downregulate the expression of TCR ζ-chain, inhibit MHC class II expression, lead to G0-G1 cell cycle arrest by inhibition of cyclin D3 and cyclin-dependent kinase (CDK4), and induce T-cell apoptosis [170,172,173,174,175]. Furthermore reactive oxygen species (ROS) level can be increased in the form of superoxide anion (O_2_^−^) by MDSCs through the upregulation of NADPH oxidase (NOX2), which can react with NO to form peroxynitrite (ONOO^-^), a strong superoxide that abrogate antigen-specific response and migration in CD8+ T-cells and CTLs [176,177]. This increase in oxidative stress within the TME by MDSCs contributes to both the immunosuppressive environment and prevention of MDSC differentiation into non-suppressive myeloid cells [178,179]. In addition, high levels of ROS and peroxynitrite has been shown to be associated with T-cell deactivation by downregulating the TCR ζ-chain expression and chemically modifying the TCR through nitrosylation, and by excluding T-cell infiltration by nitration of CCL2 (N-CCL2), which has been found to trap T lymphocytes in the stroma that surrounds the tumour and prevent their infiltration into the tumour core [180]. In general, elevated ROS levels are toxic to cells; however, MDSCs have endogenous protection from oxidative stress regulated by the transcription factor Nrf2, mitigating the toxicity caused from both the environmental and intracellular-generated ROS [181]. L-tryptophan is catabolised to produce kynurenine-based bioproducts by upregulation of indole amine 2,3 dioxygenase (IDO), a STAT-3 dependent mechanism. Consequently, the reduction of L-tryptophan and production of kynurenine have been shown to induce anergy and apoptosis in both T-cells and NK cells, and drive the differentiation of CD4+ T cells to Tregs [182,183,184,185,186]. IDO has also been implicated in the recruitment of CD4+ CD25+ Treg cells into the primary tumour and lymph nodes in breast cancer [153,187]. 

MDSCs express high levels of PD-L1 to restrain anti-tumour T-cell response. This upregulation of PD-L1 expression has been associated with the S100A9 inflammatory protein and HIF1α [167,188]. In addition, overexpression of PD-L1 has also been reported to induce aberrant hematopoiesis [188]. Another mechanism employed by MDSCs to suppress T-cell activity is through the recruitment of Tregs by the expression of immune stimulatory receptor CD40 on MDSCs [87]. Pan et al. reports that CD40 is necessary for MDSCs to both inhibit T-cell proliferation via the ligation of the ligand CD40L on T-cells and recruit Tregs [87]. Finally, MDSCs can also express immunosuppressive cytokines such as TGFβ to inhibit NK cell cytolytic activity by reducing IFNγ production [74] or IL-10 to regulate T-cell phenotype and activity [189].

**Figure 5:**
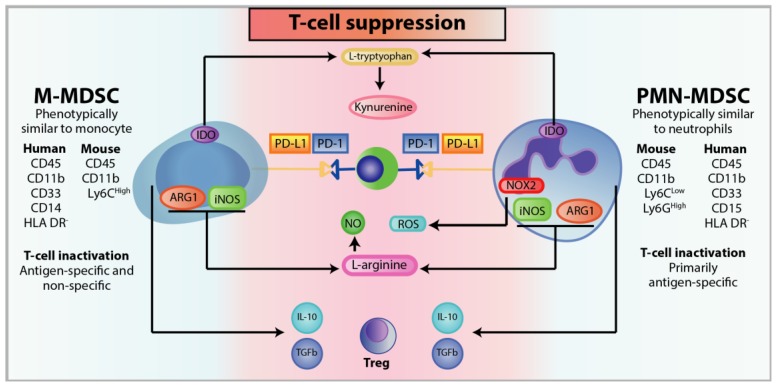
Mechanisms of T-cell suppression with phenotypic and functional differences between M-MDSC and PMN-MDSC. Both M-MDSC and PMN-MDSC display different cell surface markers and mechanisms for immunosuppression. Various mechanisms are used to suppress T-cell activity or induce T-cell apoptosis. (Top to bottom) L-tryptophan catabolism by IDO results in tryptophan starvation, leading to T-cell anergy, cell cycle arrest, and promotion of CD4 T-cells to differentiate into Tregs. Similarly, kynurenine, a tryptophan-derived catabolite by IDO inhibits T-cell and NK cell proliferation and promotes their apoptosis. In addition, kynurenine can bind to the aryl hydrocarbon receptor on T-cells to induce differentiation into Tregs. MDSCs can also induce T-cell exhaustion through elevated expression of PD-L1 to interact with the immune checkpoint PD-1. L-arginine is an essential amino acid that regulates T-cell cell cycle progression. Depletion of L-arginine by iNOS and ARG1 results in G0-G1 arrest in T-cells and downregulation of the TCR ζ-chain. The TCR will also undergo nitrosylation leading to impaired TCR signaling that is necessary for T-cell function. TCR nitrosylation results from high concentrations of NO, generated by iNOS catabolism of L-arginine, and ROS, a by-product of NOX2. MDSC can also recruit Tregs and induce their expansion via the secretion of cytokines such as IL-10 and TGFB.

Based on the subtype, MDSCs contribute to immunoevasion using different mechanisms to abrogate anti-tumour immunity (Figure 5) [90,190]. M-MDSC predominantly employs nonspecific T-cell deactivation through higher expression of ARG1, iNOS, and TGFβ; whereas PMN-MDSC produces elevated levels of ROS comparatively and exerts immunosuppressive functions by cell-to-cell contact with T-lymphocytes, rendering T-cells unresponsive to antigen-specific stimulation, but still reactive to nonspecific stimuli [176]. As such, the ratio of PMN-MDSCs and M-MDSCs populations is a major component in determining the primary mechanisms that will be used by MDSCs to abrogate immunosuppression. Generally, PMN-MDSCs are usually the predominant populations in most cancers [128,191]. However, preferential expansion of a particular MDSC subtype is influenced by numerous factors in the TME; for example, in prostate cancer M-MDSC populations outnumber PMN-MDSC, but this proportion is reversed in breast cancer [90,190]. Per cell, M-MDSC have been found to possess more potent suppressive activity compared to PMN-MDSC, but the overall strength of immunosuppression is governed by the GM-CSF secreted by tumours [192,193]. Tumour-infiltrated MDSC were also reported to possess more potent suppressive function compared to peripheral MDSCs [194].

## 4. Targeting MDSCs in Cancer

**Figure 6:**
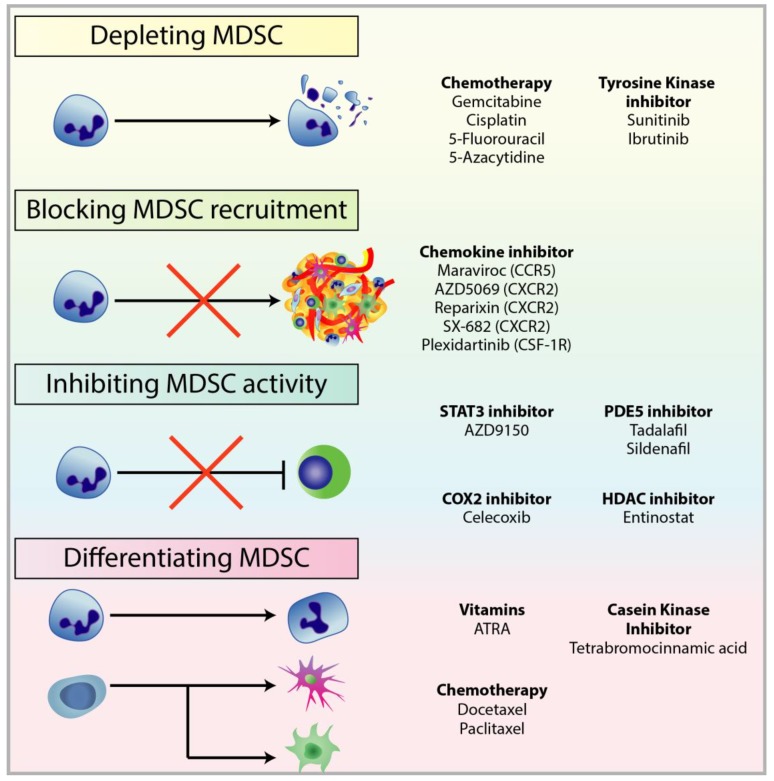
Treatments used to target different mechanisms associated with pro-tumourigenic MDSC. There are multiple therapeutic approaches against MDSC to restore anti-tumour functions in immune cells and improve immunotherapy, in particular checkpoint inhibitors. These approaches include: (1) depleting MDSC populations through low-dose chemotherapy and tyrosine kinase inhibitors; (2) preventing MDSC recruitment to the TME by targeting chemokine receptors responsible for the recruitment and migration of MDSCs; (3) attenuating the immunosuppressive mechanisms of MDSC by downregulating the expression of ARG1 and iNOS, and reducing ROS generation; (4) inducing the differentiation of MDSC into mature myeloid cells to reduce MDSC population and remove their immunosuppression.

The reduction in T-cell responsiveness by MDSCs is often associated with resistance against treatments, reducing the efficacy of immunotherapies, and ultimately in patient outcomes [55,191,195]. In breast cancer, circulating MDSCs were associated with cancer stage and metastatic burden, ultimately resulting in poor patient outcomes [72]. Clinical trials have also revealed the correlation between patient response to CTLA-4 and PD-1 checkpoint inhibitors, and the abundance of MDSC populations [150,196,197,198]. Studies on MDSCs have been more focused on assessing the dynamic roles of MDSC in immunosuppression and tumourigenesis, characterising their relationship with other cell species within the TME, and identifying new targetable pathways to deplete MDSC populations or inhibit their function [199]. MDSCs can be targeted by (1) depletion of circulating and tumour-infiltrated MDSCs; (2) prevention of MDSC recruitment and trafficking; (3) inhibition of MDSC immunosuppressive functions; and (4) differentiation of MDSCs into a non-suppressive immune state (Figure 6).

### 4.1. Depleting MDSC Populations

Low dose chemotherapy has been shown to be effective in eliminating MDSC populations in tumour-bearing mice; treatments with chemotherapy such as 5-fluorouracil (5FU), paclitaxel, cisplatin, and gemcitabine were found to deplete MDSCs and enhance anti-tumour immune activity [200,201,202,203]. However, a contrasting effect in the use of chemotherapy against MDSC was observed where MDSCs were transiently induced following cyclophosphamide treatment in tumour-bearing mice and patients [204,205].

Signalling cascades involved in MDSC expansion has also been a target in reducing the amplification of MDSC populations. For example, VEGF promotes the expansion of MDSCs, recruitment of Tregs, angiogenesis, and tumour progression. To target this, the tyrosine kinase inhibitor Sunitinib have been used successfully to deplete MDSC in patients suffering from renal cell carcinoma via blockade of VEGF and c-KIT signalling [184,206,207]. In addition, Sunitinib was found to also inhibit STAT3, and renal cell carcinoma patients treated with Sunitinib showed a reversal in MDSC accumulation and consequently T-cell suppression [206]. Finally, through a unique peptibody, Qin et al. developed a novel therapeutic peptide-Fc fusion protein that targeted the S100A family proteins to selectively deplete MDSCs without targeting other proinflammatory immune cells [208].

### 4.2. Blockade of MDSC Migration

As mentioned previously, it is strategically more effective to use therapeutic blockade to target chemokine receptors on MDSCs owing to ligand redundancy. The chemokines receptor CCR5 plays a crucial role in the chemotaxis of MDSC into the TME via the ligands CCL3, CCL4, and CCL5 [51]. However, the expression is not ubiquitous to all MDSCs; in melanoma mouse models and patients, MDSCs that express CCR5 were found to have more potent immunosuppressive mechanisms compared to the ones that do not express CCR5 [77]. Blattner et al. demonstrated that the blockade of CCR5 inhibited the recruitment and immunosuppressive activity of MDSC and improved survival in melanoma [77]. Similarly, CCR5 antagonists inhibited the metastatic potential of basal breast cancer and reduced tumour growth [209,210].

Elevated levels of CCL2 and CCL5 are present in the TME to recruit MDSC through the chemokine receptor CXCR2 [118,211]. CXCR2+ MDSC promoted tumour expansion, metastasis, EMT, and T-cell exhaustion in breast cancer [212]. By targeting CXCR2, MDSC populations were reduced and reported to decrease metastasis, promote T-cell infiltration into the tumours, improve anti-PD1 therapy, and extend survival in pancreatic cancer [159]. Additionally, CXCR2 antagonists against MDSCs have been shown to enhance the therapeutic efficacy of PD-1 immunotherapy, T-cell transfer, and chemotherapy [150,213,214]. 

CSF-1R has also been a primary target to inhibit MDSC recruitment to the tumour site to constrain tumourgenesis. CSF-1R is a tyrosine kinase receptor that when bound with its ligand CSF-1 promotes the differentiation and expansion of myeloid cells into MDSC and TAMs in addition to promoting their migration to tumours [215]. CSF-1R has been found to be upregulated in several types of cancer, such as pancreatic and breast [216,217]. Treatments targeting the receptor or its ligand CSF-1R/CSF-1 has been found to improve T-cell responses and combining CSF-1R inhibition with checkpoint blockades or adoptive T-cell transfer therapy resulted in improved anti-tumour T-cell activity and tumour regression [215,218,219]. CSF-1R inhibition and CXCR2 antagonism has also been used in combination to reduce TAM and PMN-MDSC populations and improve anti PD-1 efficacy [220]. 

Currently the following MDSC inhibitors are in clinical trials [221]: Reparixin (CXCR2) is in Phase 2 clinical trials for TNBC (NCT02370238); AZD5069 (CXCR2) is in Phase 1b/2 for advanced solid tumours and metastatic squamous cell carcinoma (NCT02499328); Plexidartinib (CSF-1R) is in Phase 2 for recurrent glioblastoma (NCT01349036); and Maraviroc (CCR5) is in Phase 1 for metastatic colorectal cancer (NCT01736813).

### 4.3. Attenuating MDSC Immunosuppressive Functions

Mitigating the potent immunosuppressive mechanisms of MDSCs have been a major therapeutic target to re-establishing T-cell activity and immunotherapy success. PGE2, as mentioned previously, is involved in inflammation, angiogenesis, tumour progression via recruitment of MDSC, and is also involved in the expression of one of the primary immunosuppressive mechanisms employed by MDSC: ARG1 [222,223,224]. Since cyclooxygenase-2 (COX-2) is upstream of the PGE2 synthesis pathway, therapies targeting COX-2, such as celecoxib, have been of great interest as a form of immunoregulatory treatment to suppress MDSC function whilst enhancing immunotherapy [225]. Disruption of the COX-2/PGE2 signalling has been successful in reducing MDSC recruitment and differentiation, repressing MDSC-associated suppressive factors such as ARG1 expression and ROS production, and shifting an inflammatory tumour profile to more anti-cancer immune rejection; consequently, COX-2 inhibition has resulted in improved CTL frequency and immune response, delayed tumour growth, and synergy between checkpoint inhibitors and dendritic cell-based immunotherapy [71,222,223,225,226].

Phosphodiesterase-5 (PDE-5) inhibitors are also able to abrogate MDSC immunosuppressive mechanisms by targeting MDSC expression and function of ARG1 and iNOS. Administration of PDE-5 inhibitors, such as sildenafil and tadalafil, have reportedly reduced inflammation in the TME, restabilised anti-tumour immune rejection through T-cell and NK cell activity, and prolonged survival in vivo [227,228,229]. Clinical trials with PDE-5 inhibitors have also shown positive results in head and neck squamous cell carcinoma and metastatic melanoma patients [230,231], abatement of MDSC and T-reg populations, enhanced intra-tumour T-cell activity, and improved patient outcome [231,232]. 

Anti-inflammatory triterpenoids have been used to activate the Nrf2 gene in MDSCs. Nrf2 is involved in modulating expression of antioxidant enzymes, including NADPH, NQO1, and hemeoxygenase, and conferring cytoprotection against oxidative stress [233]. Selective activation of Nrf2 using synthetic triterpenoids, such as CCDO-IM and CCDO-Me, has reduced intracellular ROS production (abrogating MDSC-driven immunosuppression), reduced metastasis, and has shown promising anticancer results in Phase 1 clinical trials that are well-tolerated with patients [234,235,236]. Another target to reduce oxidative stress is NO. Nitroaspirin targets iNOS to reduce ROS build-up; treatments have resulted in improved T-cell proliferation, function, invasion into the tumour core, and suppressed tumourigenesis [180,237].

STAT3 inhibition is another promising target. The antisense oligonucleotide STAT3 inhibitor, AZD9150, has been used in conjunction with immune checkpoint inhibitors in Phase 1b clinical trials for the treatment of diffuse large B-cell lymphoma. Systemic administration of AZD9150 in patients showed a marked decrease in granulocytic MDSC within the peripheral blood mononuclear cells (PBMC) [238].

### 4.4. Inducing MDSC Differentiation

Promoting the differentiation of IMC is another successful strategy in reducing MDSC populations and abolishing their immunosuppressive functions. All-trans-retinoic acid (ATRA), an agonist of retinoid receptors, inhibits retinoic signalling to shift the differentiation of MDSC into mature myeloid cells, such as macrophages and dendritic cells. ATRA treatment has resulted in reduction in T-cell suppression by directly inducing differentiation of MDSCs into mature antigen-presenting precursor cells [239]. This reduction in MDSCs and improvement in T-cell response have been observed in both mice and patients in various cancer types, such as renal cell carcinoma and small cell lung carcinoma [240,241]. The improvement by ATRA administration was reported to reduce circulating MDSC, enhance cancer vaccine treatments, improve dendritic cell function, and ameliorate antigen-specific T-cell response [240,241]. The mechanism of ATRA-induced differentiation of MDSC was reported to be mediated by glutathione synthase and neutralising ROS generation [242]. In addition, the casein kinase inhibitor tetrabromocinnamic acid was also shown to restore myeloid cell differentiation in tumour-bearing mice through improved Notch signalling [243].

Finally, epigenetic reprogramming is a novel avenue to target the pro-tumorigenic properties of MDSCs. The class I histone deacetylase inhibitor (HDAC), entinostat, has shown positive results in neutralising MDSC through epigenetic reprogramming in mouse models of pancreatic, breast, and lung cancers; and renal cell carcinoma [244,245]. Combination of entinostat with immune checkpoint inhibitors have resulted in prolonged survival, expansion of CD8^+^ T cells, and inhibition of immunosuppressive functions in both M-MDSC and PMN-MDSC via downregulation of ARG1, iNOS, and COX-2; overall, this resulted in a shift of the tumour dynamic into a more immune-susceptible TME [244,245]. Clinical trials involving entinostat are currently underway. However, clinical trials ENCORE 602 (NCT02708680) and ENCORE 603 (NCT02915523) for TNBC and ovarian cancer, respectively, have failed to increase progression-free survival. Another similar effect was observed with the use of the DNA demethylating epigenetic agent 5-azacytidine, which resulted in a reduction of MDSC and Arg1 expression [246].

Application of other chemotherapies was also reported to induce MDSC differentiation into non-immunosuppressive cell types. For example, docetaxel had a novel chemoimmunomodulatory effect by inhibiting STAT3 phosphorylation and polarising MDSC differentiation into M1-like macrophages [247]. Comparably, paclitaxel was also reported to reduce MDSC populations by promoting MDSC differentiation into dendritic cells that were independent of TLR-4 [248]. 

## 5. Combining MDSC-Targeted Treatments with Immunotherapy

**Figure 7:**
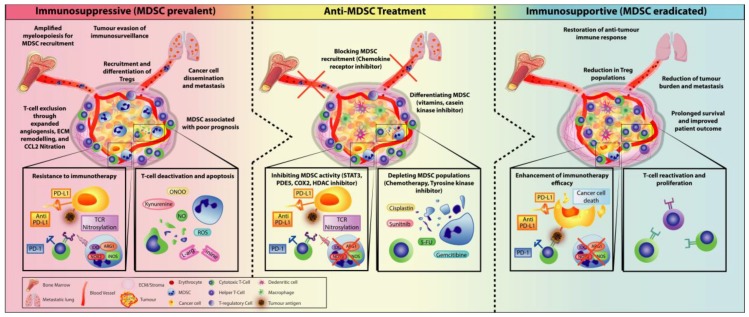
Treatment of MDSC to alleviate an immunosuppressive environment as an approach to enhancing immunotherapeutic treatments by shifting towards an immunosupportive TME. The immunosuppressive TME is propagated by various suppressive cells such as MDSCs and Tregs. Recruitment of MDSC within the TME can promote tumour expansion through various mechanisms (developing a pre-metastatic niche to help cancer cell metastasis, inducing resistance to immunotherapy by preventing the infiltration of T-cell into the tumour, suppressing and deactivating T-cell function, and inducing T-cell apoptosis) and recruitment of Tregs to further amplify immunosuppression. Thus, MDSC is often associated with poor prognosis in patients. Anti-MDSC treatments have become a major clinical target to re-establish immune control against cancer. By creating an immunosupportive environment, T-cell activity is restored, which leads to improved immunotherapy efficacy. Overall, this has resulted in prolonged survival and reduction of metastasis and tumour regression.

To improve the success of immunotherapy, there has been a paradigm shift—both the innate and adaptive layers of the immune system are simultaneously targeted to alleviate the immunosuppressive TME and re-elicit the anti-tumour response [67]. As MDSCs are one of the primary immunosuppressive cells acting as an escape mechanism for cancer cells by subverting immunosurveillance and abrogating T-cell activity, treatment strategies have been shifting towards a combination of both targeting MDSCs and immunotherapy. Indeed, targeting MDSCs may be key in diminishing tumour expansion and resensitising tumours to immune governance, thus overcoming MDSC-driven immunosuppression (Figure 7). Targeting myeloid populations alone is often insufficient as an immune-based monotherapy; however, there is compelling research and clinical trials that have shown promising results for combination therapy.

### 5.1. Checkpoint Inhibitors Combined with MDSC Depletion

Pre-clinical studies have demonstrated successful results when combining checkpoint inhibitor treatment with MDSC depletion. Kim et al. showed that co-treatment using the epigenetic modulatory drugs, entinostat and 5-azacytidine, with checkpoint inhibitors, anti-PD1 and anti-CTLA4, resulted in complete tumour regression and metastatic progression in the aggressive TNBC model 4T1, with over 80% survival rate 100 days post-implantation of the tumour [249]. Interestingly, when entinostat and 5-azacytidine were used together but not in combination with checkpoint inhibitors and vice versa, the primary tumours and metastasis remained, pointing to the synergistic effects of combination therapy in targeting MDSC and immune checkpoints. Similar results were observed in murine models of lung and renal cell carcinoma [244]. In an HER2 transgenic breast cancer and a metastatic pancreatic cancer mouse model, the entinostat-driven inhibition of MDSC activity with checkpoint inhibitor treatment resulted in an upregulation of granzyme B-producing CD8^+^ T-cells and improved the infiltration and function of adaptive immune cells. Tumour-free survival was significantly improved in these highly aggressive cancer types [245].

### 5.2. Immunotherapy Combined with Obstructing MDSC Trafficking Therapy

CXCR2+ MDSC were found to promote immune suppression when migrated to the TME; the efficacy of checkpoint inhibition in a mice model of rhabdomyosarcoma was severely limited by MDSC [150]. Disruption of CXCR2-mediated migration in MDSC was demonstrated to significantly improve anti-PD1 treatments. CXCR2+ MDSCs were also found to have potent immunosuppressive properties in human paediatric sarcoma, and thus CXCR2 may serve as a target to prevent MDSC recruitment to improve immunotherapeutic intervention. Furthermore, targeting CXCR2 improved T-cell infiltration and when combined with anti-PD1 treatment, mice bearing pancreatic cancer showed significantly extended survival [159]. SX-682, a small molecule CXCR1 and CXCR2 inhibitor, was reported to substantially reduce PMN-MDSC trafficking and infiltration to the tumour in mice [214]. Reduction in intratumour PMN-MDSC populations enhanced the accumulation of both endogenous T-cells and T-cells from adoptive transfer. Similarly to epigenetic agents, SX-682 had little anti-tumour effect as a monotherapy, but in combination therapy with checkpoint inhibitors and adoptive T-cell transfer therapy, it greatly enhanced their efficacy by inhibiting the recruitment of tumour-infiltrated CXCR2+ PMN-MDSCs [214]. SX-682 has been tested in conjunction with Pembrolizumab in P1 clinical trials for metastatic melanoma (NCT03161431).

## 6. Concluding Remarks

The identity of MDSCs is highly controversial as they can only be functionally defined. Thus, it is unsurprising that the phenotypic heterogeneity in the MDSC population had led to ambiguity in their description and characterisation between investigators, an issue that is compounded by a lack of specific markers in both mouse and human MDSCs [54,67]. MDSC are typically defined as immature myeloid cells and, during the carcinogenic process, the combination of markers expressed in MDSCs are reflective of the diversity of the myeloid lineage, which are also influenced by the type of cancer and specific TME. The definition of the phenotypic markers that encompass functional changes is vital in evaluating MDSCs’ role in tumour progression and immune evasion [131]. The application of newly developed high-throughput single-cell multi-omics techniques to understand the phenotypic and functional composition of the MDSC population will contribute to unraveling MDSC diversity and defining effective markers. 

In summary, MDSCs play a vital role in promoting tumour progression, metastasis, and creating an immunosuppressive TME; in addition, their role in resistance against immunotherapy makes them a promising therapeutic target. As we continue to develop our understanding on the characterisation and clinical value of MDSC, more selective anti-MDSC therapies will emerge. Currently, research has demonstrated the value of targeting MDSC populations as part of a combination therapy to enhance the potency of immune checkpoint inhibitors and other forms of immunotherapy. This strategy was shown to be effective in reducing tumour burden and metastasis, to the extent of improving overall survival. As such, we are now beginning to see the critical role that MDSC plays in determining patient response to treatments and their outcomes. Targeting these cells may be the key to development of a next generation of immunotherapies with improved therapeutic outcomes.

## Figures and Tables

**Table 1:** Markers used to identify MDSC populations and functions in animal models.

Mouse Marker	M-MDSC	PMN-MDSC	Notes	References
CCR2	+(high)	+	Involved in MDSC recruitment and expansion. Upregulated in MDSCs for multiple cancer types	[75,76]
CCR5	+	+	Involved in MDSC expansion and activation. Upregulated in MDSCs in melanoma.	[77,78]
CD1b	+	−	High expression of CD1b used by NKT to target MDSC for anti-tumour immunity.	[79]
CD11b (Mac-1)	+	+	Transmembrane glycoprotein for leukocyte adhesion and migration. Commonly used in combination as CD11b+, Gr-1+, Ly6C+ or Ly6G+ for identifying MDSC.	[80]
CD11c	−	−	Marker used to differentiate dendritic cells	[81]
CD38	+	+	Associated in early myeloid differentiation, activation, and migration. High expression may be associated with immature MDSC and stronger T-cell suppression	[82]
CD39	+	+	Surface ectonucleotidase that is paired with CD73 and involved in the adenosine-pathway to inhibit T-cell and NK-cell activity. Upregulated in Lewis lung carcinoma and melanoma.	[83,84]
CD40	+	+	Immune stimulatory receptor that suppresses T-cell activation, tumour specific T-reg expansion by MDSC, CXCR5-induced expansion of MDSC, and MDSC accumulation by facilitating apoptosis resistance. Upregulated in MDSC for collagen-induced arthritis, colitis, and gastric cancer	[85,86,87,88]
CD43	Unknown	+	Involved in neutrophil recruitment. Upregulated in PMN-MDSC in mammary carcinoma model.	[89]
CD45	+	+	Leukocyte common antigen used early in FACS gating to discriminate between tumour cells and immune cells.	[90]
CD49d (VLA4)	+	−	Specific marker for M-MDSC. CD49d+ MDSC were primarily monocytic and potent suppressors of antigen-specific T-cell responses.	[91]
CD54 (ICAM-1)	+ (high)	+ (low)	Immunostimulatory molecule that binds to CD11b.	[92,93]
CD62L (L-selectin)	+	+	Homing molecule that can be used to discriminate between DC and MDSC.	[94,95]
CD71 (transferrin receptor)	+	-	Marker for early erythroid precursors and proliferation. Upregulated in subcutaneous lymphoma model.	[96,97]
CD73	+	+ (high)	Surface ectonucleotidase that is paired with CD39 and involved in the adenosine-pathway. Inhibits T-cell and NK-cell activity and expansion of MDSC. Highly expressed in PMN-MDSC. Upregulated in Lewis lung carcinoma and melanoma.	[83,84]
CD80 (B7.1)	+ (low)	+/− (low)	Ligand of CTLA-4 to inhibit T-cell activity. Upregulated in MDSC by chronic inflammation in subcutaneous lymphoma, breast, and ovarian cancer	[81,97,98,99,100]
CD86	+	+	Ligand of CTLA-4 to inhibit T-cell activity. Upregulated in MDSC by chronic inflammation in breast cancer and collagen-induced arthritis.	[85,98,99]
CD98	Unknown	+	Prognostic biomarker in different cancers and functions in cysteine transportation. May also be associated with prolonging lifespan of MDSC through mTOR signalling. Upregulated in PMN-MDSC in mammary carcinoma model.	[89,92]
CD115 (M-CSFR)	+/−	+/−	Recruits tumour-infiltrating monocytes. Upregulated in MDSC in multiple cancer types.	[101,102,103,104]
CD120b (TNFR2)	+(low)	+(low)	Involved in accumulation and activation of MDSC within the tumour.	[105]
CD124 (IL-4 receptor α)	+/−	+/−	May be implicated in T-cell suppression by MDSC and MDSC survival. Upregulated in MDSC in multiple cancer types.	[106,107,108,109]
CD162 (PSGL-1)	+	+	Affects T-cell adhesion and entry to sites of inflammation.	[110]
CD170 Syglec-F (eosinophil marker)	−	−	Eosinophilic marker used to identify new subset of Eo-MDSC in chronic Staphylococcus aureus infection.	[111]
CD244	−	+/−	Cell surface receptor expressed on NK cells, DC cells and T-cells. Upregulated in MDSC in multiple cancer types.	[102,103,112]
CD279 (PD-L1)	+	+	Inhibitory ligand that suppresses T-cell activation. Upregulated in MDSC in colitis and multiple cancer types.	[81,113,114]
CX3CR1	+	+	Involved in MDSC recruitment and expansion. Can be recruited by CCL26 that are secreted by hypoxic cancer cells. Expression levels can change based on tumour progression.	[115,116]
CXCR1	+	+	Involved in MDSC recruitment and expansion. Upregulated in MDSC in multiple cancer types.	[56,117]
CXCR2	+	+	Involved in MDSC recruitment and expansion. Upregulated in MDSC in multiple cancer types.	[56,117,118]
CXCR4	+	+	Involved in MDSC recruitment and expansion. Upregulated in MDSC in multiple cancer types.	[119]
F4/80	+/−	−	Marker used to differentiate macrophages and M-MDSC.	[97,120]
Gr-1	+ (low)	+ (high)	Recognises epitope in both Ly6C and Ly6G	[80]
Ly6C	+ (high)	+ (low)	Differentiation antigen expressed in M-MDSC, macrophages, and dendritic cell precursors.	[81]
Ly6G	−	+ (high)	Differentiation antigen expressed in PMN-MDSC, neutrophils, monocytes, and granulocytes.	[81]
Mac-2 (galectin-3)	+ (high)	+ (low)	Recruits MDSC via GM-CSF pathway and induces apoptosis in T-cell via TIM-3.	[121,122]
MHC Class I	+	+	Important role in antigen processing and presentation for the activation of adaptive immunity. Expressed in both subsets of MDSC.	[123]
MHC Class II	+/− (low)	+/− (low)	MHC Class II expression varies based on disease context and mouse model used. Usually low expression or similar to tumour-free mice.	[124,125]
Sca-1, Ly6A/E	+	+	Marker for hematopoietic stem cells. Expression can be highly variable.	[97,126]
VEGFR	+	+	Receptor for VEGF, which stimulates angiogenesis and recruits MDSC. MDSC-expressing VEGFR possesses stronger immunosuppressive activities compared to other MDSCs in ovarian cancer.	[127]

**Table 2:** Markers used to identify MDSC populations and functions clinically.

Human Marker	M-MDSC	PMN-MDSC	Notes	References
CCR2	+ (high)	+	Involved in MDSC recruitment and expansion. Upregulated in MDSC in multiple cancer types, such as breast, ovarian, gastric, and melanoma.	[128,129]
CXCR4	+	+	Involved in MDSC recruitment and expansion. Upregulated in MDSC in ovarian cancer patients.	[130]
CD11b	+	+	Transmembrane glycoprotein for leukocyte adhesion and migration. Used as a myeloid marker similar to CD33.	[131]
CD13	+ (low)	+ (high)	Myeloid marker involved in cell motility.	[132,133]
CD14	+ (high)	−	Differentiation antigen expressed in M-MDSC, macrophages, and dendritic cell precursors.	[134]
CD15	−	+	Differentiation antigen expressed in PMN-MDSC, neutrophils, monocytes, and granulocytes	[81]
CD16 (FcyR)	+ (high)	+/− (low)	Discriminating antigen to exclude PMN-MDSC. Can be used to separate immature MDSC (CD16−) from PMN-MDSC (CD16+) in whole blood.	[135]
CD33	+ (high)	+ (low)	Myeloid marker that is more highly expressed in M-MDSC and dimly expressed in PMN-MDSC	[131]
CD34	+ (high)	+ (low)	Marker for hematopoietic progenitor cells used to discriminate immature MDSC.	[70,123,136]
CD38	+	+	Associated with poor prognosis. Advanced stages in cancer patients have been found to have expansion of CD38+ MDSC in head and neck, and colorectal cancer.	[82,137]
CD39	+	+	Surface ectonucleotidase that is paired with CD73 and are involved in the adenosine-pathway. Inhibits T-cell and NK-cell activity and exerts tumour cell protection against chemotherapy; for example, rapamycin. Upregulated in ovarian cancer and NSCLC.	[138,139]
CD45	+	+	Leukocyte common antigen used early in FACS gating to discriminate between tumour cells and immune cells.	[102,103,112]
CD62L (L-selectin)	+	+	Homing molecule involved in MDSC circulation. Lower expression in MDSC compared to neutrophils. Found in renal cell carcinoma patients.	[140]
CD66b	-	+	Differentiation marker expressed in PMN-MDSC.	[131]
CD68	+	−	Macrophage specific marker used to discriminate between TAM and M-MDSC	[131,141]
CD73	+	+	Surface ectonucleotidase that is paired with CD73 and is involved in the adenosine-pathway. Inhibits T-cell and NK-cell activity and exerts tumour cell protection against chemotherapy; for example, rapamycin. Upregulated in ovarian cancer and NSCLC.	[138,139,142]
CD80	+/−	−	Activation marker and ligand of CTLA-4 to inhibit T-cell activity. Expression can vary/no expression. Upregulated in advanced melanoma patients and breast cancer patients.	[80,143,144]
CD83	+/−	−	Marker used for mature dendritic cells. Can also be expressed in B and T lymphocytes. Has functions in immune cell activation and suppression	[143,144,145]
CD86	+/−	−	Activation marker and ligand of CTLA-4 to inhibit T-cell activity. Upregulated in breast cancer patients.	[142,144]
CD115 (M-CSFR)	+/−	+/−	Recruits tumour-infiltrating monocytes. Found in MDSC subset similar to precursor myeloid cells.	[146]
CD117 (cKIT)	+/−	+	Granulocyte-monocyte progenitor marker. Upregulated in colorectal cancer.	[146,147]
CD124 (IL-4 receptor α)	+	+	May be implicated in T-cell suppression by MDSC and MDSC survival. Expression can greatly vary depending on disease type.	[106,135,146,148]
CD163	+	−	Macrophage specific marker used to discriminate between TAM and M-MDSC	[131,141]
CXCR1	+	+	Involved in MDSC recruitment and expansion. Upregulated in MDSC in multiple cancer types.	[149,150]
CXCR2	+	+	Involved in MDSC recruitment and expansion. Upregulated in MDSC in multiple cancer types.	[149,150]
HLA-DR	−	−	Important role in antigen processing and presentation.	[67,81]
Lin	+/− (low)	+/− (low)	MDSC are generally negative or have very low expression for mature cell lineage markers.	[67]
VEGFR	+ (low)	+ (low)	Receptor for VEGF, which stimulates angiogenesis and recruit MDSC. Upregulated in patients with renal cell carcinoma.	[151]
